# Using Temporal Sampling to Improve Attribution of Source Populations for Invasive Species

**DOI:** 10.1371/journal.pone.0065656

**Published:** 2013-06-03

**Authors:** Sharyn J. Goldstien, Graeme J. Inglis, David R. Schiel, Neil J. Gemmell

**Affiliations:** 1 Marine Ecology Research Group, School of Biological Sciences, University of Canterbury, Christchurch, New Zealand; 2 National Institute of Water and Atmospheric Research, Aquatic Biodiversity and Biosecurity, Christchurch, New Zealand; 3 Centre for Reproduction and Genomics, Department of Anatomy, University of Otago, Dunedin, New Zealand; 4 Allan Wilson Centre for Molecular Ecology and Evolution, University of Otago, Dunedin, New Zealand; National Institute of Water & Atmospheric Research, New Zealand

## Abstract

Numerous studies have applied genetic tools to the identification of source populations and transport pathways for invasive species. However, there are many gaps in the knowledge obtained from such studies because comprehensive and meaningful spatial sampling to meet these goals is difficult to achieve. Sampling populations as they arrive at the border should fill the gaps in source population identification, but such an advance has not yet been achieved with genetic data. Here we use previously acquired genetic data to assign new incursions as they invade populations within New Zealand ports and marinas. We also investigated allelelic frequency change in these recently established populations over a two-year period, and assessed the effect of temporal genetic sampling on our ability to assign new incursions to their population of source. We observed shifts in the allele frequencies among populations, as well as the complete loss of some alleles and the addition of alleles novel to New Zealand, within these recently established populations. There was no significant level of genetic differentiation observed in our samples between years, and the use of these temporal data did alter the assignment probability of new incursions. Our study further suggests that new incursions can add genetic variation to the population in a single introduction event as the founders themselves are often more genetically diverse than theory initially predicted.

## Introduction

The theory of invasion genetics has been discussed in the literature for many decades [1], but advanced molecular tools have only been applied to invasion ecology within the last 25 years [1]–[3]. Allozyme markers were initially used to investigate genetic diversity between invasive and native populations within invaded regions [4]–[6]. Subsequently, the 21^st^ Century saw increased use of PCR tools to assess the relationship between the genetic structure and the global geographic distribution of aquatic invasive species such as the Mediterranean fan worm *Sabella spallananzii* [7] and riverine invaders *Gammarus fossarum* and *Dreissena polymorpha* [8]. Numerous molecular studies have now been done on a wide variety of invasive species, the purpose of which was primarily to identify species and source populations, potential vectors and invasion pathways [3].

The primary and most conclusive outcome from the application of molecular tools has been the identification of cryptic species [3], [9]–[12]. In contrast, of the many studies aiming to identify the source populations of invasions, very few have been able to do so due to admixture, multiple sources and secondary introductions [13]–[18]. For instance, the most extensively studied invasive marine species, the European green crab *Carcinus maenas* shows some clear genetic affinities for non-native populations in Tasmania and Nova Scotia but studies also highlight the veiling of source populations and genetic affinities due to multiple incursions (in North American populations) and/or unsampled source populations (in Japanese populations) [16], [19]. Molecular examination of another crustacean, *Caprella mutica*, also identified multiple pathways of introduction to coastal regions throughout the Atlantic, from Asia, Europe and America [20]. Exceptions to this pattern of multiple introductions include the invasive alga *Codium fragile* spp. *tomentosoides*, shown to have two distinct introductions into Europe [21], and the Pacific acorn barnacle *Balanus glandula*, for which independent incursions to Japan and Argentina were identified [22]. Part of the difficulty is that assignment of an incursion to a particular source requires good representation from the range of potential source populations, with the problem of missing populations well-described [23]. However, temporal sampling of genetic structure is equally important in populations at the invasion front, where high propagule pressure and rapid population turnover occur.

The contemporary evolution of invasive species has also been investigated over the past decade. Unfortunately, many of these studies have been initiated long after the first incursion and this ‘snapshot’ of genetic diversity has been used as a starting point to decipher the many possible mechanisms that may be driving the observed patterns. For example, *Undaria pinnatifida* [17], *Rapana venosa* [24], and *Styela plicata* [25] have all been transported around the world for more than 70 years with relatively few recent introductions recorded. In contrast, a study of *Carcinus maenas*, was conducted over an eight year period with large sample sizes and good historical data [26]. This study showed how temporal data can highlight the interplay between environmental conditions and natural dispersal in the regional spread of an introduced species along a coast from its point of introduction. *C. maenas* clearly showed asymmetric dispersal with new haplotypes working into existing populations and homogenising coastal populations through time [26].

New incursions, or recently established populations, may provide further insights into contemporary evolution. The initial period of invasion, which includes both founders and their progeny, is crucial for assessing the contribution of individuals to the persistence of the population and for predicting the evolutionary trajectory of the population. At this stage of the incursion, with relatively few colonisers, genetic changes are likely to occur as a result of stochastic processes in small populations [27] and the spatial dynamics of the newly formed and diverging population. The occurrence of rare alleles and genetic heterozygosity likely depend primarily on the effective population size at initial incursion and population growth, as alleles that persist through the genetic bottleneck become more common within the expanding population, and so large shifts in allelic frequency could be expected [1], [27], [28]. In addition, it is likely that, for invasive species, temporal variation in allele frequencies will result from the mixing of distant, genetically structured, populations from different sources; a Wahlund effect is known to occur when cohorts are mixed as a single population over time [29], [30]. An increase in heterozygote deficiency and departure from Hardy-Weinberg equilibrium is expected under a temporal Wahlund effect as the number of pooled cohorts increases [31]. A similar effect should occur if invading populations were sourced from different locations over time. A recent study of an invasive ascidian *Perophora japonica* [32] in Europe, very nicely showed a reduction in genetic diversity over a 9-year period, beyond the initial bottleneck, with subsequent differentiation among European populations.

**Figure 1:**
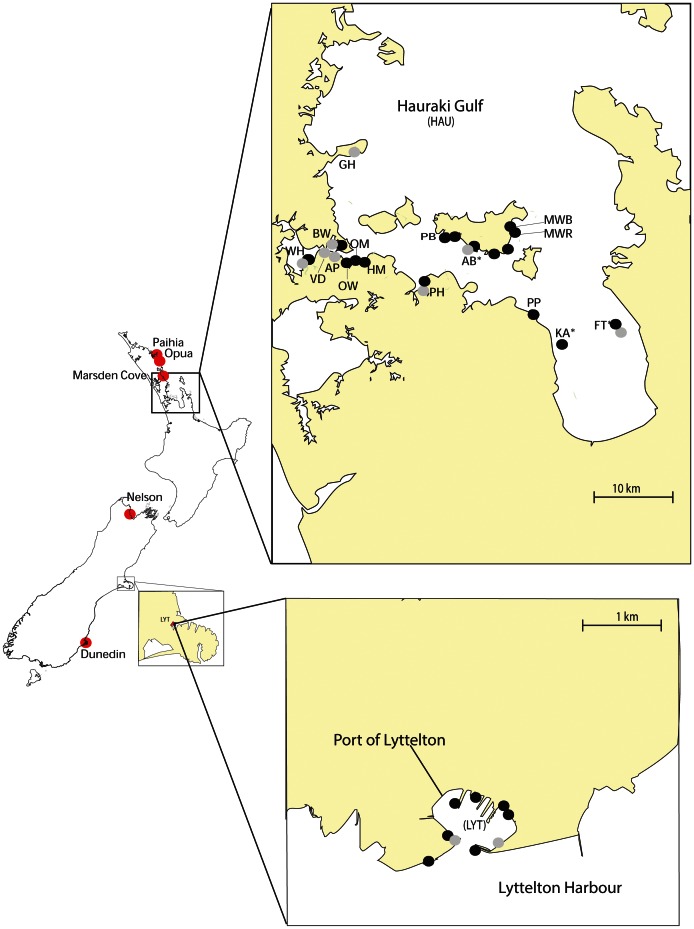
Sampling sites of *Styela clava* throughout New Zealand in 2006 (grey) and 2007 (black). Abbreviations: AB, Awaawaroa Bay; AP, Auckland Port; BW, Bayswater marina; FT, Firth of Thames; GH, Gulf Harbour marina; HM, Half-Moon Bay marina; KA, Kaiaua; MW, Man-of-War Bay; O, Orapiu wharf; OM, Orakei marina; OW, Orakei Wharf; P, Puatiti Point; PB, Putiki Bay; PH, Pine Harbour marina; VD, Viaduct marina; WH, Westhaven marina; Port of Lyttelton (LYT). * Depicts populations sampled within aquaculture farms. Red circles represent locations of new incursions sampled in this study. Modified from Goldstien *et al.* [35].

New Zealand is unique in that many relatively recent marine incursions have been well-documented as a result of regular monitoring of ports and marinas by the Ministry of Agriculture and Forestry, Biosecurity New Zealand (now the Ministry for Primary Industries - MPI). One recent and increasingly wide-spread ascidian invader, *Styela clava*, was first recorded in New Zealand in 2005 in widely separated populations of the North (Hauraki Gulf) and South Islands (Lyttelton, Fig. 1). It has subsequently spread to secondary locations throughout the country. This species is abundant in the northern Hauraki Gulf where it has been recorded at densities of up to 100 individuals per m^2^ and occurs in much lower numbers of between one to 10 individuals per m^2^ in the southern port of Lyttelton Harbour [33]. Previous studies on *S. clava* have provided a global and regional snapshot of the genetic diversity and population connectivity for this species, which shows that vessel activity is a major vector for the regional spread of *S. clava* in New Zealand and Britain [14], [34], [35]. Unlike *C. maenas*, which is known to disperse naturally over long distances, *S. clava* relies more heavily on anthropogenic transport for regional spread [35] and, therefore, exchange among populations should be more easily traced to internal movement among marinas or ongoing input from overseas ports. Here, we examined two common components in the application of genetics to the understanding of invasive species: 1) the utility of genotypic and haplotypic data when assigning new incursions, 2) The accuracy of genetic assignments with different levels of temporal and spatial sampling.

## Materials and Methods

### Sample Collections

To address the aims of the study we collected *Styela clava* individuals from hard surfaces within the top metre of the subtidal zone. Three levels of collections were made during 2007, and the data generated from this study were then compared with data collected from 2006 in two previous studies [14], [35] (Fig. 1).

1. We obtained 46 specimens of *Styela clava* collected either from the hull of boats arriving to port (PAH), or from marinas where newly documented incursions had occurred (MAR, DUD, NEL, OPU), herein referred to as new incursions. These incursions were sampled during targeted surveillance and boat hull inspections in 2007, undertaken by the National Institute of Water and Atmospheric Research (NIWA) and MPI (Fig. 1) and were not found in previous surveillance inspections at these marinas.
2. In April 2007 we collected 171 *Styela clava* individuals from four locations in the Hauraki Gulf and one location in the Port of Lyttelton that were also sampled in a 2006 study [35].

To expand the spatial resolution of the dataset, in April 2007 we collected an additional 197 individuals from a further 10 locations throughout the Hauraki Gulf and Lyttelton Harbour. These locations were not covered in the 2006 study by Goldstien *et al.* [35] as the 2006 data set was focused on marinas and aquaculture farms, excluding the natural habitat studied here. These additional sites are not considered new incursions as *S. clava* was recorded from these locations preceding the 2006 study. No specific permits were required for the described field studies. No specific permissions were required for the locations or activities as collections were not on privately-owned land and did not involve endangered or protected species.

All specimens were preserved in 70% ethanol for storage. DNA extractions, mitochondrial DNA sequencing (new incursions only) and microsatellite genotyping (all samples) were done using the protocols of Goldstien *et al.* [35]. To confirm genotypes and avoid technical biases in the data, we also genotyped 30 individuals collected in 2006 [35] alongside these new samples. Six of the eleven microsatellites developed for *S. clava* [36] were used in this study (1A9, 2H9, 1D11, 2B12, 1H1, and 1C8). The remainder of the microsatellites did not achieve consistent amplification across the dataset and so were not used in this study

### New incursion analyses – mitochondrial and microsatellite data

We used mtDNA and genotypic data to investigate the equality of these molecular markers in accurately assigning new incursions. Data from previous studies on the global distribution of mtDNA haplotypes and microsatellite genotypes [14], [35] were compared using the assignment of new incursions sampled in this study. The relationship of haplotypes was assessed using statistical parsimony networks constructed in TCS [37], incorporating all haplotypes previously identified from New Zealand, Australia and North America [14]. Further statistical tests were not performed on these data due to the small and imbalanced sample sizes. Genalex6 [38] was used to assign microsatellite genotypes of the new incursions. New incursions were treated as an unknown population in the assignment analysis.

### Microsatellite analyses

All individuals sampled in 2007 were genotyped for comparison to locations sampled in 2006 and to investigate the role of temporal sampling in the assignment of the new incursions. Diversity indices such as expected heterozygosity (H_e_) and allele frequency for loci and populations were estimated using Genalex6 [38], and a chi-squared test was run for each locus within each population, to assess Hardy-Weinberg equilibrium. To determine the allelic richness (Ar) of populations, which accounts for differences in sample sizes [39], we ran rarefaction in HP-RARE 1.0 [40], with rarefaction set to a sample size of 13. We then used t-statistics to test for differences in Ar among years. To compare H_e_ & F_IS_ statistics between 2006 and 2007 samples we used the randomisation procedure [41] in Fstat [41].

F_ST_ and pairwise distance statistics [42], [43] were calculated using Arlequin v. 3.1 [44] and from these data we assessed the degree to which the five 2007 populations represented a sample of the five 2006 populations. To do this we applied statistics more common to the study of species diversity, whereby each individual was treated as a sample unit and the presence of alleles recorded for each individual across multiple loci was treated as species abundance. Species accumulation curves were done in Primer v.6 (Primer-E Limited, 2009) where the Chao2 index was compared against observations (S_obs_). Due to the occurrence of rare alleles, Chao's Jaccard estimator (Chao-Jacc-Est) of abundance-based similarity index [45]–[47], which accounts for “unseen individuals’ based on rare alleles was used and compared against the Jaccard and Bray-Curtis indices to estimate the similarity between 2006 and 2007 samples, using EstimateS v. 7.5 (Colwell R.K). Finally, using chi-squared analyses we assessed the efficacy of using datasets with and without temporal variation to assign the new incursions.

## Results

### New incursion analyses – similarities between genotypes and haplotypes

**Figure 2:**
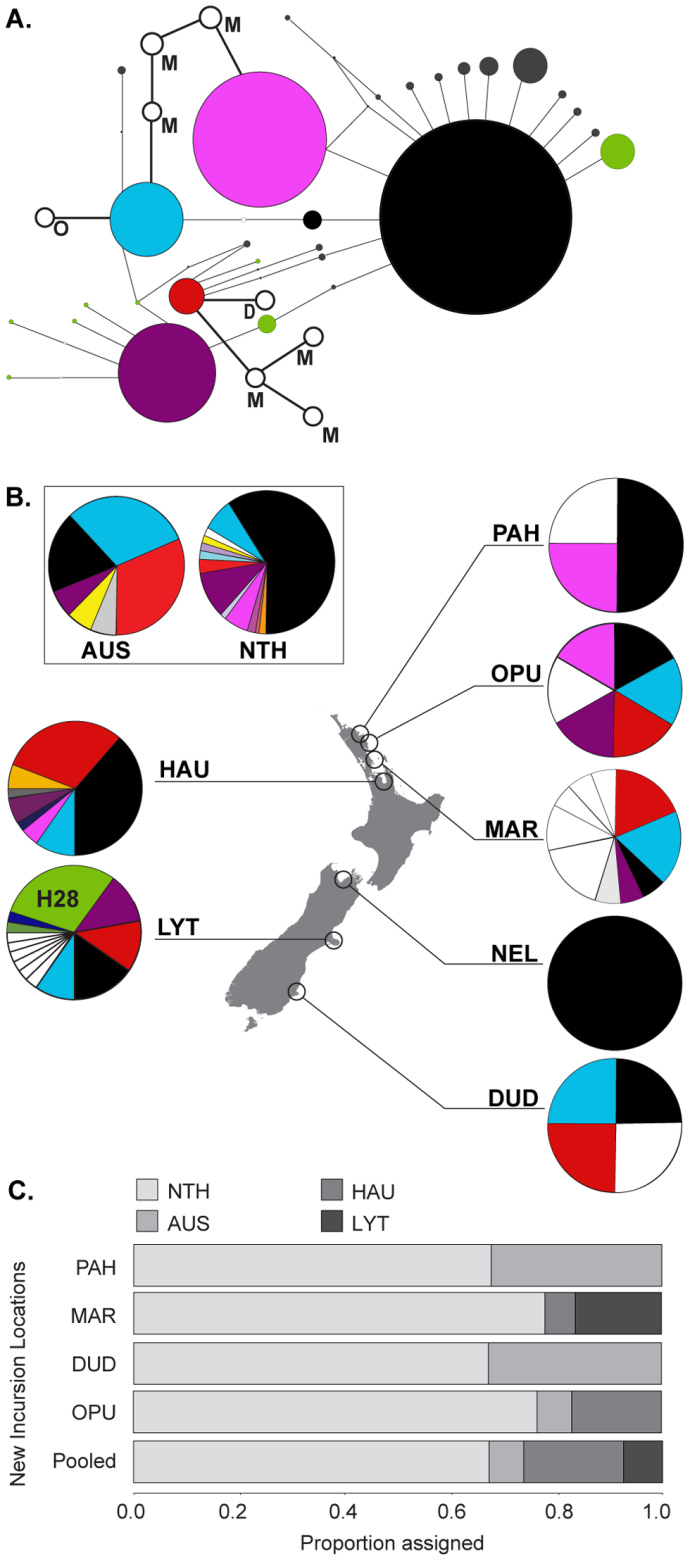
Haplotypic and genotypic data for the new incursions of *Styela clava* sampled in 2007: (A) The haplotypes unique to Marsden Cove (M), Opua (O) and Dunedin (D) are shown relative to their relationship with haplotypes recorded in 2006: The six haplotypes shared with data obtained in 2006 (colours match locations in figure 2B); the haplotypes present in locations outside of New Zealand (grey); and those present only in Lyttelton (green). Circles represent haplotypes and the size reflects the frequency of each haplotype within the data set. (B) The distribution and frequency of haplotypes present in the new incursion sampled in 2007 and in the populations sampled by Goldstien *et al.* (2010) in 2006. The colours are consistent with 2A. Pies represent the frequency of each haplotype within the populations. (C) The proportion of genotypes within the new incursion populations assigned to populations sampled by Goldstien et al. (2010) in 2006. Abbreviations: PAH, Paihia; OPU, Opua; MAR, Marsden Cove; DUD, Dunedin; NEL, Nelson; LYT, Lyttelton; HAU, Hauraki Gulf; AUS, Australia; NTH, North America. 2A & B are modified from Goldstien *et al.* (2010 and Goldstien *et al.* (2011).

The haplotypic and genotypic data obtained were consistent in their ability to identify new incursions. Eight new and six previously assigned mtDNA haplotypes were observed in the new incursions (Fig. 2a). However, a haplotype previously found to be unique to Lyttelton, and occurring in high frequency there (H28), was not observed in any of the new incursions (Fig. 2b). In addition, unique haplotypes identified from new incursions at Marsden Cove, Opua and Dunedin were closely related to haplotypes previously found in multiple populations, including in the North and South Islands of New Zealand [14] (Fig. 2a). Alleles were consistently amplified from six microsatellite loci for a total of 46 individuals of *Styela clava* sampled from four marinas and one boat hull in 2007. Considering the low sample size for these new incursions, the genotypic diversity (allelic richness and heterozygosity) was high relative to populations previously sampled from New Zealand (Table 1). Genotypic assignment of each of the new incursions consistently showed a high proportion of assignment (>60%) to North America (Fig. 2c) and only one population, Marsden Cove, showed a proportion of assignment to the Port of Lyttelton. Marsden Cove was also the most diverse site with six of the eight unique haplotypes. When all individuals from the new incursions were pooled, the assignment remained consistent with the separate groupings.

**Table 1:** Descriptive statistics for genotypic data sampled from *Styela clava* populations throughout New Zealand.

Site		Year sampled	Habitat	n	N_all_	A_r_	F_IS_	H_E_
**Lyttelton**	LYT	*2006*	*Port*	*13*	*31*	*2.71*	*0.27*	*0.62*
		2007	Port	70	58	2.87	0.29	0.65
**Hauraki Gulf (HAU)**								
Awaawaroa Bay	AB	*2006*	*Oyster farm*	*49*	*45*	*2.85*	*0.27*	*0.63*
		2007	Oyster farm	27	41	2.87	0.26	0.62
Bayswater	BW	*2006*	*Marina*	*19*	*34*	*2.74*	*0.22*	*0.61*
		2007	Marina	32	49	3.00	0.22	0.66
Firth of Thames	FT*	*2006*	*Mussel farm*	*22*	*36*	*2.85*	*0.32*	*0.62*
		2007	Mussel farm	26	38	2.93	0.30	0.64
Westhaven	WH	*2006*	*Marina*	*29*	*40*	*2.90*	*0.26*	*0.65*
		2007	Marina	16	27	2.70	0.34	0.54
Half Moon Bay	HM	2007	Marina	26	35	2.87	0.30	0.64
Kaiaua	KA*	2007	Mussel Farm	27	36	2.70	0.28	0.66
Orakei	OM	2007	Marina	22	32	2.72	0.15	0.58
Orapiu Wharf	OW	2007	Wharf	6	22	2.78	0.29	0.57
Puatiti Point	PP	2007	Intertidal reef	5	17	2.56	−0.02	0.56
Man of War Bay	MWB	2007	beach	26	42	3.04	0.16	0.66
Man of War Bay	MWR	2007	Intertidal reef	24	39	2.92	0.07	0.66
Putiki Bay	PB	2007	Intertidal reef	11	27	2.74	0.10	0.56
Putiki Bay	PB*	2007	Oyster farm	29	40	2.97	0.29	0.65
TeMatuku Bay	TM*	2007	Oyster farm	21	36	2.70	0.22	0.58
**New Incursions**								
Pahia	PAH	2007	Hull	6	21	2.94	0.35	0.59
Nelson	NEL	2007	Marina	1	9	1.50	-	0.43
Marsdon Cove	MAR	2007	Marina	19	36	3.55	0.18	0.62
Dunedin	DUD	2007	Marina	11	32	3.56	0.08	0.62
Opua	OPU	2007	Marina	9	29	3.48	0.28	0.88

Genetic characteristics are represented across six polymorphic loci and include: Site location, year sampled, habitat type, sample size (n), number of alleles (Nall), allelic richness (Ar), fixation index (F_IS_) and gene diversity (H_E_). Note: Italicised text highlights the five populations sampled in 2006 and 2007.

* highlights aquaculture farm populations.

### 2007 microsatellite analyses

Alleles were consistently amplified from six loci for a total of 368 individuals of *Styela clava* sampled from 15 populations in 2007 (Table 1). In addition, 30 individuals sampled in 2006 were re-genotyped to assess possible shifts in allele peaks due to changes in the instrument and running procedure. Four loci showed no shift in allele size, while two were adjusted for a shift of one base pair for data acquired across different years. All loci were polymorphic and significant genetic structure was observed between Hauraki Gulf and Lyttelton populations (F_ST_, 0.106; P<0.01). The number of alleles per locus ranged from 8 (Sc2B12) to 19 (Sc1H1) (Table S1). Chi-square tests showed significant departures from Hardy-Weinberg equilibrium for 13 of the 16 sampled populations (with adjusted nominal level for multiple randomisations). Two populations from natural reef environments (PP and PB), comprising two small rock platforms, and Westhaven Marina showed no significant difference from H-W equilibrium for any of the loci sampled. For all other populations, two to four of the six loci were out of H-W equilibrium. There was no significant difference in the observed genetic diversity among sites. The greatest diversity was observed in populations from Bayswater Marina (A_r_, 3.00; H_e_, 0.54; Table 1) and Man-of-War beach (A_r_, 3.04; H_e_, 0.66; Table 1). The lowest genetic diversity was observed in a small intertidal reef population at Puatiti Point (A_r_, 2.56; H_e_, 0.56; Table 1), within Hauraki Gulf.

### Temporal genetic change (2006/2007)

**Figure 3:**
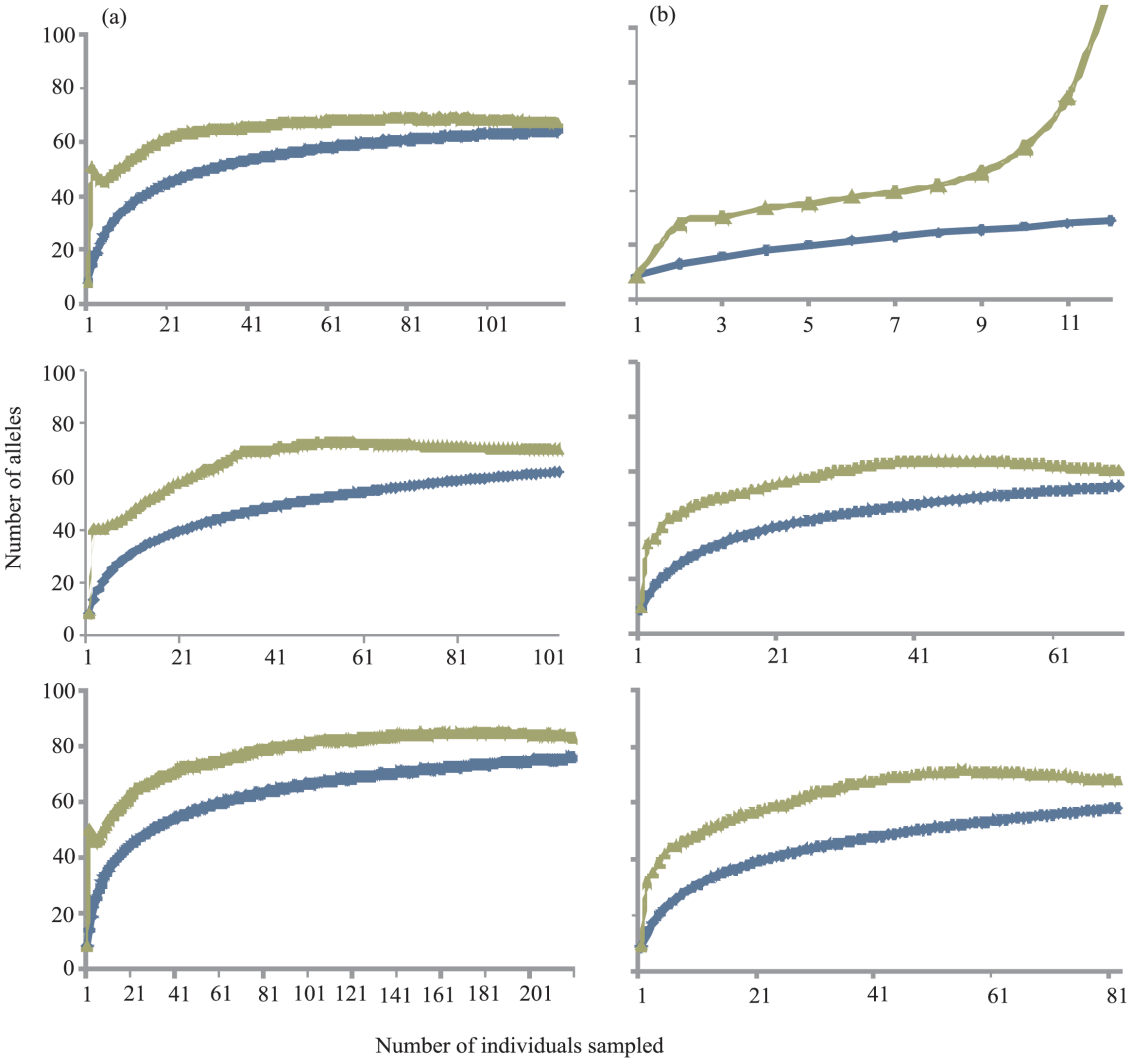
Allele accumulation curves for observed data (green) and the Chao2 index (blue). Populations represent Hauraki Gulf (a) and Lyttelton Port (b), from 2006 (top graph), 2007 (middle) and years combined (bottom graph).

Five of the populations sampled in 2006 were re-sampled in 2007 to investigate the genotypic stability over this two-year period (Table 1). Comparisons between years for these five populations showed no significant difference for inbreeding index (F_IS_), allelic diversity (Ar), or heterozygosity (H_E_). Species (allele) accumulation curves show that 85–92% of the diversity estimated by the Chao2 index was captured in each year for the Hauraki Gulf (Fig. 3a) and 25–93% for Lyttelton (Fig. 3b). However, a plateau of allelic abundance was not observed in any of the data sets despite having sampled over 100 individuals in the Hauraki Gulf, indicating a relatively high allelic richness with a large proportion of singleton and doubleton alleles. The estimated similarity between populations was high when accounting for “unseen alleles” (Chao-Jacc-est) but was considerably lower for the classic Jaccard and Bray-Curtis estimates that do not account for “unseen alleles” (Table 2).

**Table 2:** Allele frequencies for *Styela clava* populations sampled in 2006 and 2007.

Population		N_all_	N_06_	N_07_	n/d	CJ	J	BC
Bayswater	BW	53	34	49	19/5	0.6	0.5	0.1
Firth of Thames	FT*	43	36	38	7/7	0.9	0.5	0.6
Awaawaroa Bay	AB*	53	45	41	8/12	0.6	0.4	0.4
Westhaven	WH	42	40	27	2/15	0.6	0.4	0.4
**Hauraki Gulf**		**76**	**64**	**62**	**12/14**	**0.8**	**0.7**	**0.7**
**Lyttelton Port**	**LP**	**44**	**29**	**39**	**15/5**	**1.0**	**0.5**	**0.3**

Genetic characteristics are represented across six polymorphic loci and include: Site location with sample ID, total number of alleles (Nall), number of alleles for 2006 (N_06_) and 2007 (N_07_) and the ratio of alleles new in 2007 : alleles dropped in 2007 (n/d), Chao2-Jaccard-est index (CJ), Jaccard classic (J), Bray-Curtis Index (BC).

In the Lyttelton population, 15 ‘new’ alleles were observed in 2007, of which only three were not observed in any other populations. Of the 29 alleles observed in Lyttelton in 2006, only five were not observed in the populations in 2007, but all of these did occur in the Hauraki Gulf populations (Table 2). Input of new alleles to populations within the Hauraki Gulf was high for one marina population but loss of alleles was more common in other populations. However, when all populations within the Hauraki Gulf were pooled in each year, the similarity estimates increased and the proportion of new and dropped alleles reduced, suggesting spatial shifts in allele frequencies (Table 2). There was no significant difference between years.

### New incursion assignment efficacy with different data sets

**Figure 4:**
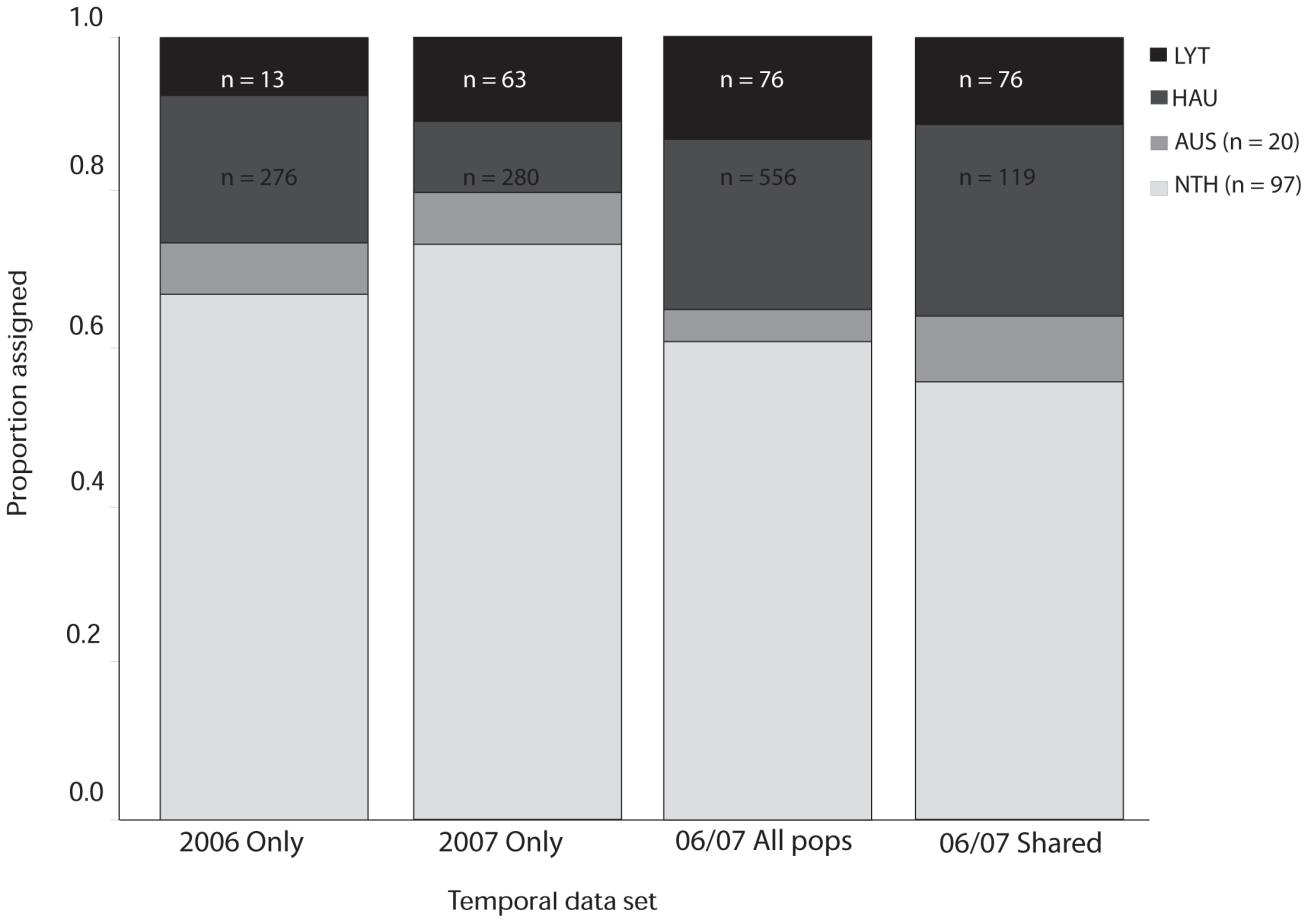
Proportional assignments of *Styela clava* individuals sampled from new incursions. Stacked bars show the proportion of individuals with the highest probability of genetic assignment to known populations in New Zealand (NZ) and overseas. Data sets were analysed separately for year-sampled combinations: 2006 and 2007 separately; combined 2006 and 2007 for all populations sampled (06/07 All pops) and the five populations sampled in 2006 and 2007 (06/07 Shared). Samples from North American and Australia were included in all assignment analyses. Abbreviations: LYT, Lyttelton Port; HAU, Hauraki Gulf; NTH, North America; AUS, Australia. Sample size (n) for each data set is shown.

Genotypic assignment of individuals from new incursions showed a low probability of assignment to New Zealand populations sampled in 2006 and 2007 (Fig. 4), although no single source could be assigned to these new incursions. The combined data from 2006 and 2007 showed a significant difference in the proportion of new incursions assigned to the northern Hauraki Gulf populations and the southern Lyttelton Port populations compared with the separate 2006 and 2007 data sets, suggesting their assignment probabilities depend on the temporal aspect of the data sets (Fig. 4; X^2^ = 49.95, p<0.01). In all cases the new incursions were assigned with high probability to populations sampled in North America (0.67, 0.73, 0.61, 0.57 for the 2006, 2007 data, all individuals from 2006 and 2007 and the 2006/2007 shared populations data sets respectively). For all four of these data sets, the Hauraki Gulf had the highest number of individuals sampled, yet this does not appear to affect the results obtained. Temporal sampling increased the assignment to New Zealand, regardless of whether using only the shared populations or the whole data set, suggesting that this is not a function of increasing the sample size.

## Discussion

The results obtained here highlight the importance of temporal genetic sampling and the application of baseline genetic data sets in determining the origin and spread of new incursions. To our knowledge this is the first study to use existing genetic data to assign individuals to sources as they arrive at new locations within a region. The key findings of this study are: 1) new incursions recorded in New Zealand exhibit high mitochondrial and nuclear genetic diversity and both markers were consistent in their assignment of individuals to populations; 2) genetic diversity has been maintained over a two-year period within approximately 10 years of the initial introduction; 3) significant spatial structure between the North and South Island populations is maintained over the two-year period, despite extensive regional admixture and population growth; 4) temporal sampling was important in the accurate assignment of new incursions.

*Styela clava* is transported around the globe in biofouling on vessels and relies heavily on anthropogenic transport for international and domestic spread [14]. Unlike the very clear picture of asymmetric dispersal displayed by the crab *Carcinus maenas* along the coast of Nova Scotia [26], the continuous translocation of *S. clava* makes for a very “chaotic” pattern of genetic admixture and allelic shifts. Models of maritime transportation networks have highlighted how complexity in the transport pathways, coupled with stochastic demographic events (e.g., recruitment to a vessel and establishment in a new location) can drive an unpredictable sequence of invasion from primary and subsequent incursions, with potential for considerable re-assortment of populations of frequently transported species [48]–[50]. Although it is expected that allelic change and genetic drift are directly related to population growth and demographic stochasticity [27], [28], it is apparent that the stochasticity and temporal stability of transport pathways may be more influential in maintaining temporal stability of alleles in introduced species. For instance, the Hauraki Gulf experiences high levels of recreational vessel traffic. *Styela clava* is much more likely to be transported by recreational vessels than merchant vessels (because the former sit idle for longer, are less well maintained and travel at slower speeds), and this is reflected in the admixture observed among populations and between years in our data. In contrast, the Port of Lyttelton receives a high number of merchant vessels but much less recreational traffic, which is reflected in the significant genetic differentiation between the Lyttelton and Hauraki Gulf populations, maintained over the two-year period, or about four generations for *Styela clava* [51].

New incursions for *Styela clava* were not resolved to specific source locations in this study, but they were proportionally assigned to northern and southern New Zealand populations, or to overseas populations for which data were available. The pooling of data from populations sampled in 2006 and 2007 significantly changed the proportion of assignments to northern and southern New Zealand from 20% and 25% to 35% and 37%. The change observed with the temporal approach could simply be a function of increasing the sample size and capturing more alleles. However, using only the shared populations actually decreased the sample size for the Hauraki Gulf. In addition, the data collected from 2006 and 2007 showed no significant difference and all alleles were captured in both datasets, albeit the distribution of these alleles did vary within years. It is more likely that there is a trade-off occurring here. For instance, the 2006 data for Hauraki Gulf includes the Port of Auckland, marinas and aquaculture farms; in 2007 Auckland Port was not sampled, but the marinas and aquaculture farms were sampled, as well as additional sites from natural habitats. By combining the 2006 and 2007 data, we have effectively sampled more individuals of the important populations around marinas and aquaculture farms, while the more minor sites have been eliminated. Analysis of this core data set, with a strong element of temporal resampling, indicates that temporal sampling may enhance the accuracy and likelihood of assignment to the true source region as unsampled (rare) alleles in the population may be important to assignments, particularly if these change in frequency from year-to-year.

The new incursions studied here highlight a key aspect of founding populations and the interplay of transport pathways and population dynamics in the genetic diversity of new incursions. Numerous genetic studies have shown that genetic diversity in populations of invasive species is higher than expected in founder populations under bottleneck conditions [52]. Several authors have definitively put this “paradox” to rest, highlighting that the gap between time since introduction and the genetic study, in most cases, would likely need no more than the temporal shifts and genetic divergences expected from growing populations to explain the diversity observed [53], [54]. Our data are unique in that we have sampled populations within an estimated 20 generations from the initial incursion, as well as new incursions within the first generation of establishing within a location. From these first generation samples, it is clear that the diversity of the founding populations is not necessarily low; indeed some founders exhibit greater diversity than many of the established populations, and provide a diverse genetic pool for regional translocation, without invoking rapid population growth or multiple introduction processes.

These data also provide a powerful model for further monitoring the change in genetic diversity with population dynamic shifts over time. Several other studies have successfully used historical genetic data to investigate genetic change over time [55]. observed large genetic variance and temporal fluctuations in allele frequencies of the introduced fly *Rhagoletis completa* using samples taken from the initial founding population and again 30 years after its initial introduction. Similarly, hybrid zones of *Mytilus* spp. along the coast of California were sampled in 1994–95 and again in 2005–07 showing a large range shift toward the equator in the subtropical species *M. galloprovincialis* following a decade of climatic oscillation, and Peres-Portela [32] showed a reduction of diversity in a population established from a single introduction.

Perhaps genetic equilibrium is rarely met in introduced species, particularly at small initial population sizes, where an individual's contribution to the next generation is amplified and is critical to successful establishment and population growth. However, the importance of temporal variation in allele frequencies may differ between species with short- and long-generation times, with different per capita rates of growth (*r*) from high or low fecundity species, and between marine and terrestrial invaders. For example, iteroparous species may have more temporal buffering of reproductive success because they are spreading the risk of failure over multiple reproductive events and years, as seen in the sea urchin *Paracentrotus lividus* [56]. Greater temporal variation may also be expected in populations of invaders where adults have life-histories adapted to broadcast spawning and high planktonic mortality and stochasticity, combined with disturbances experienced during the invasion process [57], [58].

Increasing the genetic database for invasive species to include spatial and temporal variation of populations could prove to be an invaluable tool for pre-border management of NIS. Currently, genetics is predominantly used as a snapshot tool to identify species and their origin, yet there are very few instances where the pathway has been identified successfully from this one-off sampling approach. While much criticism has been focused on the lack of inclusion of source populations and spatial variation in genetic sampling, very little attention has been given to the change in genetic signature with time through the invasion process. Our work suggests that addressing the variability of source populations and the potential for genetic drift in small populations with the use of temporal sampling may be a critical element in the future use of genetic tools for invasive species management.

## Supporting Information

Table S1 Allele frequencies for *Styela clava* populations sampled in 2006 and 2007.
(DOCX)
