# Supplementary material for: Using Temporal Sampling to Improve Attribution of Source Populations for Invasive Species
Source: PLoS One. 2013 Jun 3;8(6):e65656. doi: 10.1371/journal.pone.0065656 (PMC3670837; doi:10.1371/journal.pone.0065656)
Supplement: Table S1 — Allele frequencies for Styela clava populations sampled in 2006 and 2007. (DOCX) [file pone.0065656.s001.docx]

**Supplementary Table S1**. Allele frequencies for *Styela clava* populations sampled in 2006 and 2007.

| **Locus** | **Allele** | **BAY6** | **BAY7** | **MF6** | **MF2** | **OYST6** | **OYST8** | **WHM6** | **WHM7** | **LYT6** | **LYT7** |
| --- | --- | --- | --- | --- | --- | --- | --- | --- | --- | --- | --- |
| 1A9 | 131 |  |  |  |  | 0.010 |  |  |  |  |  |
|  | 133 |  | 0.016 | 0.048 |  | 0.020 |  |  |  |  | 0.021 |
|  | 141 | 0.263 | 0.234 | 0.238 | 0.220 | 0.194 | 0.385 | 0.214 | 0.200 | 0.346 | 0.042 |
|  | 147 |  | 0.031 |  |  |  |  |  |  |  |  |
|  | 153 |  |  |  |  | 0.010 |  |  |  |  |  |
|  | 155 | 0.053 |  | 0.024 |  | 0.020 | 0.019 | 0.018 |  |  | 0.063 |
|  | 157 |  |  |  | 0.060 |  | 0.019 |  |  |  |  |
|  | 159 | 0.237 | 0.359 | 0.310 | 0.340 | 0.378 | 0.308 | 0.482 | 0.433 | 0.077 | 0.104 |
|  | 161 | 0.026 |  | 0.024 |  |  | 0.019 | 0.018 |  | 0.077 | 0.021 |
|  | 163 | 0.026 |  |  |  |  |  |  |  |  | 0.063 |
|  | 165 |  | 0.016 |  |  |  |  |  |  |  | 0.042 |
|  | 167 | 0.026 | 0.047 |  | 0.020 | 0.020 | 0.019 | 0.054 |  | 0.154 | 0.188 |
|  | 169 | 0.211 | 0.203 | 0.190 | 0.260 | 0.143 | 0.135 | 0.161 | 0.333 | 0.346 | 0.458 |
|  | 171 | 0.105 | 0.063 | 0.143 | 0.080 | 0.143 | 0.058 | 0.036 |  |  |  |
|  | 173 | 0.026 | 0.031 | 0.024 | 0.020 | 0.051 |  | 0.018 |  |  |  |
|  | 179 |  |  |  |  |  | 0.038 |  | 0.033 |  |  |
|  | 181 | 0.026 |  |  |  | 0.010 |  |  |  |  |  |
| 2H9 | 273 |  | 0.078 | 0.159 | 0.135 | 0.051 | 0.058 | 0.017 | 0.067 |  |  |
|  | 275 | 0.250 | 0.344 | 0.523 | 0.423 | 0.582 | 0.596 | 0.397 | 0.567 | 0.192 | 0.354 |
|  | 277 | 0.444 | 0.281 | 0.182 | 0.231 | 0.245 | 0.269 | 0.241 | 0.267 | 0.385 | 0.583 |
|  | 279 | 0.222 | 0.172 | 0.045 | 0.115 | 0.071 |  | 0.172 | 0.033 | 0.346 | 0.042 |
|  | 281 | 0.028 | 0.031 | 0.045 |  | 0.010 |  | 0.069 | 0.033 |  | 0.021 |
|  | 283 | 0.028 | 0.047 |  |  | 0.020 |  | 0.034 |  | 0.038 |  |
|  | 285 |  |  | 0.023 | 0.019 | 0.010 | 0.019 |  |  |  |  |
|  | 291 | 0.028 | 0.047 | 0.023 | 0.077 | 0.010 | 0.038 | 0.069 | 0.033 | 0.038 |  |
|  | 293 |  |  |  |  |  | 0.019 |  |  |  |  |
| 1D11 | 202 |  |  | 0.045 |  | 0.020 |  | 0.069 |  |  |  |
|  | 238 |  | 0.016 |  |  |  |  |  |  |  |  |
|  | 292 |  | 0.016 | 0.045 |  | 0.020 | 0.038 | 0.052 |  | 0.100 | 0.050 |
|  | 306 |  | 0.016 |  |  |  |  |  |  |  |  |
|  | 324 |  | 0.032 |  | 0.019 |  | 0.019 |  |  | 0.100 |  |
|  | 326 |  | 0.129 | 0.023 | 0.058 | 0.061 | 0.173 | 0.034 |  | 0.200 | 0.125 |
|  | 328 | 0.211 | 0.161 | 0.068 | 0.442 | 0.163 | 0.115 | 0.224 | 0.250 | 0.100 | 0.175 |
|  | 330 | 0.447 | 0.097 | 0.250 | 0.192 | 0.245 | 0.135 | 0.310 | 0.250 | 0.100 | 0.150 |
|  | 332 | 0.079 | 0.274 | 0.250 | 0.096 | 0.133 | 0.135 | 0.034 | 0.125 | 0.400 | 0.325 |
|  | 334 | 0.132 | 0.032 | 0.068 | 0.096 | 0.143 | 0.077 | 0.121 | 0.094 |  | 0.100 |
|  | 336 | 0.132 | 0.097 | 0.182 | 0.096 | 0.122 | 0.231 | 0.121 | 0.281 |  | 0.050 |
|  | 340 |  | 0.129 | 0.068 |  | 0.092 | 0.077 | 0.034 |  |  | 0.025 |
| 2B12 | 116 |  | 0.016 |  |  |  |  |  |  |  |  |
|  | 120 | 0.026 | 0.016 |  |  |  |  |  |  |  | 0.109 |
|  | 126 | 0.921 | 0.938 | 0.977 | 0.860 | 0.949 | 0.981 | 0.904 | 0.964 | 0.962 | 0.826 |
|  | 132 |  | 0.016 |  |  |  |  |  |  |  |  |
|  | 134 | 0.053 | 0.016 | 0.023 | 0.140 | 0.051 | 0.019 | 0.096 | 0.036 | 0.038 | 0.065 |
| 1H1 | 218 |  |  |  |  |  |  |  |  |  | 0.021 |
|  | 230 | 0.053 | 0.016 | 0.067 | 0.058 | 0.010 | 0.019 | 0.034 | 0.107 |  |  |
|  | 232 |  |  |  |  |  |  |  |  |  | 0.042 |
|  | 236 |  | 0.016 |  |  |  |  |  |  | 0.038 | 0.021 |
|  | 238 | 0.079 | 0.016 | 0.100 | 0.096 | 0.104 | 0.130 | 0.121 | 0.143 |  |  |
|  | 240 |  | 0.016 |  |  | 0.021 |  | 0.017 |  |  |  |
|  | 242 |  |  |  | 0.019 |  |  |  |  |  |  |
|  | 244 | 0.026 | 0.094 | 0.100 | 0.096 | 0.083 | 0.056 | 0.017 |  |  | 0.042 |
|  | 246 |  | 0.016 |  | 0.058 |  | 0.019 |  |  |  | 0.021 |
|  | 250 |  |  |  | 0.077 |  | 0.056 |  |  |  | 0.104 |
|  | 252 | 0.342 | 0.156 |  | 0.115 | 0.260 | 0.278 | 0.172 | 0.286 | 0.231 | 0.208 |
|  | 254 | 0.105 | 0.172 | 0.200 | 0.135 | 0.135 | 0.130 | 0.086 | 0.107 | 0.077 |  |
|  | 255 |  |  |  |  | 0.010 |  |  |  |  |  |
|  | 256 | 0.342 | 0.391 | 0.400 | 0.308 | 0.323 | 0.259 | 0.431 | 0.357 | 0.500 | 0.292 |
|  | 258 |  | 0.094 |  |  |  |  |  |  | 0.077 | 0.167 |
|  | 266 |  |  |  |  |  | 0.019 |  |  |  |  |
|  | 272 |  |  |  |  | 0.010 | 0.019 | 0.017 |  | 0.038 | 0.063 |
|  | 274 | 0.053 | 0.016 | 0.133 | 0.038 | 0.031 | 0.019 | 0.069 |  | 0.038 | 0.021 |
|  | 276 |  |  |  |  |  |  | 0.034 |  |  |  |
|  | 2556 |  |  |  |  | 0.010 |  |  |  |  |  |
| 1C8 | 107 |  |  |  |  |  |  |  |  |  | 0.042 |
|  | 109 | 0.333 | 0.240 | 0.273 | 0.327 | 0.378 | 0.426 | 0.364 | 0.344 | 0.192 | 0.375 |
|  | 111 | 0.583 | 0.360 | 0.545 | 0.462 | 0.347 | 0.278 | 0.341 | 0.469 | 0.577 | 0.563 |
|  | 113 | 0.083 | 0.300 | 0.091 | 0.173 | 0.224 | 0.167 | 0.205 | 0.031 | 0.192 | 0.021 |
|  | 115 |  |  |  |  |  |  |  | 0.031 |  |  |
|  | 117 |  | 0.040 | 0.091 | 0.038 | 0.051 | 0.130 | 0.091 | 0.125 | 0.038 |  |
|  | 127 |  | 0.040 |  |  |  |  |  |  |  |  |
|  | 129 |  | 0.020 |  |  |  |  |  |  |  |  |
